# The Association of Peer Smoking Behavior and Social Support with Quit Success in Employees Who Participated in a Smoking Cessation Intervention at the Workplace

**DOI:** 10.3390/ijerph16162831

**Published:** 2019-08-08

**Authors:** Floor A. van den Brand, Puck Nagtzaam, Gera E. Nagelhout, Bjorn Winkens, Constant P. van Schayck

**Affiliations:** 1 Department of Family Medicine, Maastricht University (CAPHRI), 6229 HA Maastricht, The Netherlands; 2 Department of Health Promotion, Maastricht University (CAPHRI), 6229 HA Maastricht, The Netherlands; 3 IVO Research Institute, 2595 AA The Hague, The Netherlands; 4 Department of Methodology and Statistics, Maastricht University (CAPHRI), 6229 HA Maastricht, The Netherlands

**Keywords:** smoking cessation, workplace, employees, financial incentives, social support, peer support, social environment

## Abstract

The current study investigated whether quit success among employees who participated in a smoking cessation intervention at the workplace was associated with social support from, and the smoking behavior of, people in their environment. Tobacco-smoking employees (*n* = 604) from 61 companies participated in a workplace group smoking cessation program. Participants completed questionnaires assessing social support from, and the smoking behavior of, people in their social environment. They were also tested for biochemically validated continuous abstinence directly after finishing the training and after 12 months. The data were analyzed using mixed-effects logistic regression analyses. Social support from colleagues was positively associated with 12-month quit success (odds ratio (OR) = 1.85, 95% confidence interval (CI) = 1.14–3.00, *p* = 0.013). Support from a partner was positively associated with short-term quit success (OR = 2.01, 95% CI = 1.23–3.30, *p* = 0.006). Having a higher proportion of smokers in the social environment was negatively associated with long-term abstinence (OR = 0.81, 95% CI = 0.71–0.92, *p* = 0.002). Compared to having a non-smoking partner, long-term quit success was negatively associated with having no partner (OR = 0.48, 95% CI = 0.26–0.88, *p* < 0.019), with having a partner who smokes (OR = 0.40, 95% CI = 0.24–0.66, *p* < 0.001), and with having a partner who used to smoke (OR = 0.47, 95% CI = 0.26–0.86, *p* = 0.014). In conclusion, people in a smoker’s social environment, particularly colleagues, were strongly associated with quit success. The workplace may, therefore, be a favorable setting for smoking cessation interventions.

## 1. Introduction

Current smoking cessation therapy focusses mainly on the individual smoker who wants to quit, although smoking is a social behavior that is greatly influenced by a smoker’s social environment [1,2,3]. Smoking cessation group therapy, where groups of individual smokers quit together, is designed to stimulate peer support and peer pressure in order to improve the quit success of participants [4]. However, during most smoking cessation treatments, the wider social environment of the quitter, including family, friends and colleagues, is usually not actively involved. This is unfortunate, since social support from, and the smoking behavior of people in the social environment may be key factors in quit success [2,5,6].

Longitudinal studies have shown evidence that smokers with a partner who smokes are less likely to attempt to quit and to quit successfully, while having a non-smoking partner can promote quitting [6,7]. Smokers who live in a household with a larger proportion of smokers are less likely to quit smoking [8,9] and smokers who have more smokers among their friends are less likely to attempt to quit and to succeed in a quit attempt [2,10]. The social environment can also promote quitting smoking, for example if smokers perceive that their community has strong anti-smoking norms [11] or if smokers are motivated to quit by people close to them [12]. The negative influence of other smokers on quit success may be caused by a lack of social support for quitting smoking [13,14], more positive norms toward smoking [1] and more exposure to smoking cues during a quit attempt [15]. These results show that it can be more difficult to quit smoking for people who have many smokers in their social environment.

Apart from the home environment, the workplace can be considered as another social setting where people spend a large part of the day together, interact, and may affect each other’s beliefs and behaviors [16,17]. Frequent exposure to others smoking at work was associated with a lower likelihood of recent smoking cessation among workers in a nationally representative sample of US adults [17]. Another study hypothesized that co-workers may function as models, and found clustering effects of smoking status and the amount smoked in work groups [16]. A network analysis showed that a co-worker who quits smoking increased the likelihood of a subject’s smoking cessation by 34%. The influence of co-workers could, therefore, potentially be used in interventions to stimulate smoking cessation in the social setting of the workplace. For instance, group smoking cessation programs with colleagues can have the extra advantage of stimulating social support and peer pressure from people who are nearby for an important part of the day [18]. However, little research has been done on the potentially valuable effect of social support from colleagues on smoking cessation in a workplace intervention setting [4].

Although people in the social environment have been shown to influence smoking cessation, it is not entirely clear at what stage of the cessation process social dynamics are particularly influential. Some studies found that positive social support was associated with short-term, but not with long-term quit success, while the absence of negative support such as criticism predicted continuous abstinence [5,19,20]. Having a partner who also quits smoking at the time of a quit attempt was positively associated with smoking abstinence in older couples up to many years later [6]. It is important to examine further whether support from, and the smoking behavior of the social environment can affect only short-term or also long-term quit success, as this can inform health promotors on when social support can be effective or show when social support should be enhanced.

The current study is a secondary analysis from a cluster randomized trial, in which employees participated together with colleagues in a smoking cessation group training program at their workplace. In the current study, we investigated whether the social influence of different types of people in the environment of the participant, including colleagues who were peer quitters in the group smoking cessation program, was associated with quit success. We also assessed associations of the number of smokers among people in the close circle of the participant (family, friends, and colleagues) and the smoking status of their partner with successful smoking cessation. Additionally, we investigated whether the extent to which these persons were supportive toward the participant’s quit attempt was related to quit success. We assessed this social influence on short-term quit success directly after the smoking cessation group training program, and on long-term quit success 12 months later.

## 2. Methods

### 2.1. Design

The current study is a secondary analysis of data from a cluster randomized trial (RCT), of which the methods and results have been described in more detail in previous publications [21,22]. In short, the RCT involved 61 companies organizing a smoking cessation group training at the workplace, which consisted of seven weekly 90-minute sessions divided over a period of two months. In the RCT, 31 companies were randomized into the intervention group and 30 into the control group. In total, 604 employees participated in the RCT. In the intervention group, employees earned vouchers of €350 in total if they successfully quit smoking for 12 months. The study was registered in the Netherlands Trial Register under number NL5537. The study was approved by the medical ethical committee METC-Z, Heerlen, The Netherlands (No. 16-N-63). All participants signed for their informed consent.

### 2.2. Setting and Participants

The participants were 604 tobacco-smoking employees aged at least 18 from companies in the Netherlands. Data were collected between March 2016 and March 2018.

### 2.3. Measurements

To assess social influence, the same questionnaire was sent out at baseline, and at four follow-up measurements: directly after the smoking cessation training had ended (two months after baseline), three months, six months and 12 months after the training had ended. In case of no response, participants were called by phone or were sent a text message with a reminder to fill out the questionnaires. Participants were awarded a voucher of €25 for completing the questionnaires. Smoking abstinence was assessed directly after the training and at 12 months after the training by a research assistant who visited the study participants at their workplace or at home. The research assistant used a Smokerlyzer (Bedfont) to measure expired-air carbon monoxide (CO) concentrations.

### 2.4. Variables

The main outcome variables were continuous abstinence from smoking directly after finishing the smoking cessation program and continuous abstinence 12 months after finishing the program, confirmed by CO measurement with a threshold value of 9 parts per million. [23]. Participants with measurements above the threshold or participants who we were not able to measure were considered smokers in the analyses [23]. Among the independent variables was the number of smokers in a participant’s social environment, which was measured at baseline with the question: “How many of the five closest friends, acquaintances or colleagues that you regularly spend time with are smokers?” Response categories were 0–5.

We assessed the smoking status of the partner at baseline (T0) and directly after finishing the training (T1), using the question: “Does your partner smoke?”. Response categories were: “yes”, “no”, “I do not have a partner” or “don’t know”. A new variable was created by combining the smoking status of the partner at baseline and at the end of the cessation program. This resulted in the categories “never smoker”, “continued smoking”, and “stopped between T0 and T1”. Participants who answered “I do not have a partner” at both T0 and T1 were categorized as “no partner”. All other combinations of answers were categorized as “other”. “Don’t know” was categorized as missing.

Other independent variables were the social support that participants received from colleagues who also participated in the group smoking cessation program, colleagues who did not participate in the cessation program, their partner, and friends and family. These were assessed with the question “How supportive were (colleagues who participated in the training program/other colleagues/partner/friends and family) of your quit attempt in the last two months?” at follow-up directly after the smoking cessation program. Response categories were “don’t know”, “very supportive”, “moderately supportive”, and “a little supportive”. For the analyses, the “very supportive” category was retained. The last two response categories were combined into “not very supportive”, and the “don’t know” category was classified as missing. For the variable “social support from partner”, the response category “I do not have a partner” was combined with “not very supportive” in the multivariable analysis to prevent collinearity effects with the “I do not have a partner” response option in the questions assessing the smoking status of the partner. 

Additional covariates in the multivariable analysis were measured at baseline and included intervention group (incentive vs. no incentive), nicotine dependence (Fagerström score 0–10) [24], income level, and educational level. Income level consisted of individualized net household income and was based on tertiles. Educational level was ‘low’ for none completed, elementary school and lower secondary education; ‘moderate’ for middle secondary education; and ‘high’ for upper secondary education and university.

### 2.5. Statistical Analyses

Differences between participants lost to follow-up and participants included in the analyses were tested using independent T tests for numerical variables and Chi square tests for categorical variables. To assess the influence of the social environment on smoking abstinence, we performed separate analyses for each individual variable, correcting only for the covariates (intervention group, income level, educational level, and nicotine dependence). For the outcome “smoking abstinence after 12 months”, we included the same covariates and we additionally selected the variables with a P value lower than 0.200 and included these in a multivariable analysis, because the expected that a small number of quitters did not allow including all variables and covariates in the model. We used multivariable mixed-effects logistic regression analysis with a random intercept at company level to adjust for the clustering of participants within a company. Participants with missing data on the outcome “smoking abstinence” were considered as smokers and included in the intention to treat analyses. Other missing data were imputed using all other fixed variables that were included in the model, and values on these variables from other measuring points. We created 50 complete datasets using multiple imputation with a maximum number of 20 iterations and used trace lines to check convergence. We performed a complete case analysis as a sensitivity analysis that only included participants without any missing values. We considered two-sided *p* values ≤0.05 as statistically significant. IBM SPSS statistics for Windows (version 25.0) (Arrmonk, New York, USA) was used to compute descriptive statistics, RStudio version 1.1.383 MICE (Boston, MA, USA) package for multiple imputation, and lme4 package (glmer function) for the mixed-effects logistic regression analysis.

## 3. Results

### 3.1. Participants

The mean age of the participants was 45.1 (standard deviation (SD) 10.2) and there were more male than female participants (Table 1). The mean Fagerström score was 4.4 (SD 2.0), which indicates a moderate nicotine dependence [25]. Of the 469 participants who had a partner at baseline, 43% had a partner who smoked (result not reported in Table 1). The mean number of smokers within the participants’ close circle of five friends, family or colleagues at baseline was 2.8 (SD 1.5).

### 3.2. Loss to Follow-Up

There were no missing data on the outcome “smoking cessation”. Compared to participants who completed the follow-up questionnaire directly after finishing the smoking cessation training (*n* = 542), participants who did not complete this questionnaire (*n* = 62) more often had a low income and a higher nicotine dependence, but did not differ significantly in terms of educational level, age, sex, or intervention group. 

### 3.3. Short-Term and Long-Term Abstinence of Participants

Table 2 presents the CO-validated continuous smoking abstinence of participants. Directly after completing the smoking cessation program, 482 of 604 participants (80%) had successfully quit smoking. At 12 months follow-up, 206 of 603 participants (34%) abstained from smoking (one participant was excluded from the 12-month analysis because of unavoidable loss to follow-up according to the Russell Standard).

### 3.4. Associations of Social Support with Quit Success

Table 2 presents how supportive the social environment was during the period from the start until right after finishing the smoking cessation program. A large majority (78%) of the participants found their colleagues who also participated in the smoking cessation group training very supportive. By contrast, only 39% of the participants reported other colleagues to be very supportive. The support of friends and family was approximately equally divided between not very supportive and very supportive. Of the participants, 20% did not have a partner. Among the participants who did report having a partner, 68% (277 out of 410) found their partner very supportive of their quit attempt. Table 3 shows that having very supportive group training colleagues was significantly and positively associated with quit success directly after finishing the smoking cessation program (odds ratio (OR) = 3.63, 95% confidence interval (CI) = 2.07–6.37, *p* < 0.001), and also with 12-month quit success (OR = 1.85, 95% CI = 1.14–3.00, *p* = 0.013). The support of other colleagues (not participating in the smoking cessation program) was not significantly associated with quit success at either time. Having very supportive friends compared to not very supportive friends was not significantly associated with short-term or 12-month quit success. Finally, having a very supportive partner was positively associated with quit success (OR = 2.01, 95% CI = 1.23–3.30, *p* = 0.006), but only directly after finishing the training and not after 12 months. The results of the separate analyses of the social influence of the social environment that were performed to select variables to include in the multivariable analysis showed effects comparable to the multivariable analysis (Appendix A).

### 3.5. Associations of Smoking Behavior of Persons in the Social Environment with Quit Success

**Figure 1:**
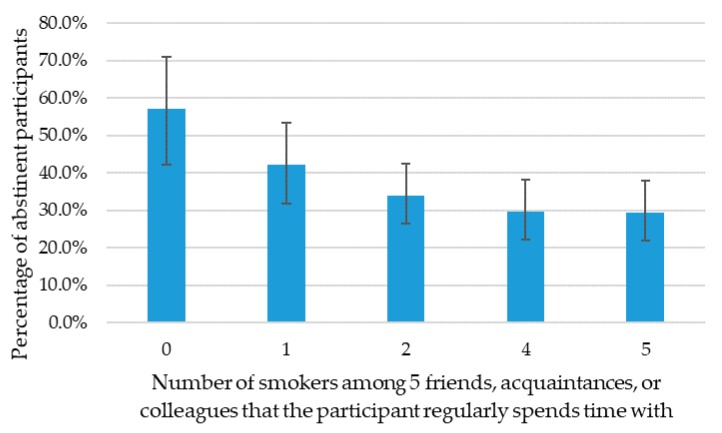
Percentage (95% confidence interval (CI)) of participants that continuously abstained from smoking up to 12 months after finishing the smoking cessation program, presented for each number of smokers among the five closest friends, acquaintances, or colleagues that the participant regularly spends time with.

Among the participants’ five closest friends, acquaintances or colleagues, the mean number of smokers was 3.8 (1.5) (Table 2). A larger number of smokers among these five persons was negatively associated with quit success (Figure 1 and Table 3). This effect was not statistically significant directly after the smoking cessation training program, but was significant 12 months after finishing the program (OR = 0.81, 95% CI = 0.71–0.92, *p* = 0.002). This result means that with each extra smoker within someone’s close circle of five people, the odds of continuous smoking cessation after 12 months decreased by approximately 19%. Of the participants who had a partner, most had a partner who had never smoked (58%), 26% had a partner who smoked and 15% had a partner who had quit smoking between baseline and the end of the smoking cessation program. The smoking status of the partner was also associated with quit success. Compared to having a partner that had never smoked, having no partner was significantly and negatively associated with quit success directly after finishing the smoking cessation training (OR = 0.46, 95% CI = 0.25–0.85, *p* = 0.014). The partner’s smoking status during the quit attempt was also significantly associated with 12-month quit success. Compared to participants who had a partner that had never smoked, all other partner categories (a partner who continued smoking, a partner who had stopped smoking, having no partner or other) were significantly and negatively associated with successful quitting. The results of the separate analyses of the smoking behavior of persons in the social environment that were performed to select variables to include in the multivariable analysis showed comparable effects to the multivariable analysis (Appendix A).

### 3.6. Sensitivity Analyses

The sensitivity analyses where only complete cases were included showed comparable results to the main analyses of the impact of the smoking behavior of and social support from people in the social environment on short-term as well as long-term smoking cessation success (Appendix B).

## 4. Discussion

In the current study, we aimed to investigate whether social support from, and the smoking behavior of colleagues, partners, and family and friends of employees who participated in a workplace smoking cessation training program were associated with short- and long-term quit success.

The results of the current study confirm our hypothesis that colleagues have an important influence on smoking cessation success. Not only did most participants (78%) find their colleagues who also participated in the smoking cessation group training very supportive, but also support from these colleagues was significantly associated with quit success. Having a very supportive partner was also positively associated with short-term quit success, while support from friends and family and other colleagues was not significantly associated with quit success. These results suggest that people who are closely involved in the quitting process of the smoker are especially important. An interesting finding was that unlike partner support, social support from colleagues who participated in the training was associated with a higher probability of smoking cessation up to 12 months after finishing the training. Previous research had suggested that social support mainly influences the achievement of abstinence, not the continuation of abstinence in the long term [5,19,20]. However, the lack of an effect of partner support on long-term quit success may also be explained because the actual smoking behavior of the partner rather than the support may be ultimately decisive for quit success. Likewise, the smoking behavior of colleagues may be particularly influential [1]. Therefore, the effect of social support from colleagues should be explored further while taking into account the smoking behavior of these colleagues. The positive association of colleague support with smoking cessation found in the current study underlines the importance of social processes, such as peer support and peer pressure, that are unique to group counselling as compared to individual treatment. This potential beneficial effect of colleagues on quit success is interesting from a health promotion perspective; it suggests that the work environment may be a promising environment for interventions, perhaps particularly for smokers in whose home environment smoking is more prevalent and socially accepted.

Our study indicated that the number of other smokers in the social environment has a large effect on the success of people who want to quit smoking. This result confirms the influence of smokers in the social environment found in previous research [1,2,20], and is comparable with an earlier study that showed an increase in relapse odds of 1.12 for each friend who smoked [26]. Additionally, that particular study found that a higher proportion of smokers among friends was associated with relapse only approximately one month after quitting, which is in line with our results showing that the number of smokers was not significantly associated with quit success directly after finishing the training, but was associated with quit success at 12 months. This may be explained because the participants feel more vulnerable in the first phase of quitting smoking and may avoid social situations that could lead to relapse, while in a later phase participants may feel more secure about their abstinence and may be less cautious. The findings of the current study imply that smoking cessation counsellors should evaluate whether there are many smokers in the quitter’s social environment and aim to overcome corresponding barriers to quit success.

According to our results and previous research [6,8], having a partner who has never smoked seems to be a factor that can prevent smoking relapse. Compared to having a partner that never smoked, having no partner was negatively associated with short-term quit success. The negative influence of not having a partner has also been shown in other studies [6,9]. An explanation could be that quitters benefit from having someone who monitors their abstinence and provides social control in the first difficult phase of quitting smoking. A partner could also help maintain a high motivation to stay abstinent. The association of the smoking status of the partner during the smoking cessation program with 12-month quit success was also prominent. Compared to having a partner who had never smoked, having no partner, having a partner who smoked, and even having a partner who had quit smoking substantially decreased the probability of quit success. These results confirm studies showing the effect of partners on smoking cessation and continuous abstinence [1,6,27]. For successful cessation treatments, it may be important to also include the partner in the quitting process.

### Strengths and Limitations

A strength of the current study is that it showed a potentially very effective source of social support that can be used to improve quit success in smokers, namely the support of colleagues who participated in a workplace group smoking cessation program alongside the smoker. Further research should explore whether the positive association of peer support with quit success can be generalized to general population settings. The current study was a post hoc analysis, which implies that research questions and hypotheses were made after the data were collected. Further research is therefore needed to confirm our findings. Another limitation of the current study is that while we investigated the influence of the smoking behavior of the social environment and social support between baseline and the end of the smoking cessation program, we did not assess the influence of changes in these variables within the remainder of the 12-month follow-up period. Furthermore, data on the smoking behavior of partners were self-reported by participants, so they may not be accurate if the participants misclassified their partner’s smoking status. However, it can be argued for the current study that the smoking status of the partner as perceived by the participant is more relevant than the partner’s objective smoking behavior. In addition, in order to determine the smoking status or change in smoking status of the partner between baseline and after finishing the smoking cessation training (a period of eight weeks), limitations in the data forced us to assume that these partners were still the same individuals, while it is possible that some participants changed partners in between. Moreover, because few participants had relapsed into smoking at the follow-up measurement directly after completion of the program, we performed separate analyses to assess the associations of the independent variables with quit success to prevent unreliable results. For the outcome “quit success directly after completion of the program”, we were, therefore, unable to determine whether variables would still be independently associated with quit success if all variables were included in the model. Finally, the amount of missing data that needed to be imputed was rather high for some variables, and there were differences in some variables between participants with and without missing values, which could indicate selection bias. However, the complete case analysis showed conclusions similar to the main analysis.

## 5. Conclusions

In the current study, social support from colleagues was strongly associated with the long-term quit success of employees who participated in a group smoking cessation training program at the workplace. Whether a quit attempt is successful may also depend on the smoking behavior of the partner and the proportion of smokers in the social environment. The current study implies that it is desirable to include the social environment in the smoking cessation process in order to increase the probability of quit success. The workplace can be a favorable setting for smoking cessation interventions, as colleagues may provide a valuable source of support.

## Figures and Tables

**Table 1:** Baseline characteristics of participants.

Characteristic	Participants
**Age (*n* = 599)**	
Mean (standard deviation (SD))	45.1 (10.2)
**Sex (*n* = 604)**	
Women	381 (37%)
Men	223 (63%)
**Educational level (*n* = 579)**	
Low	159 (27%)
Moderate	255 (44%)
High	165 (29%)
**Income level (*n* = 535)**	
Low	179 (33%)
Moderate	175 (33%)
High	181 (34%)
**Nicotine dependence (Fagerström score 0–10) (*n* = 573)**	
Mean (SD)	4.4 (2.0)
Minimally dependent (<4)	184 (32.1%)
Moderately dependent (4–6)	305 (53.2%)
Highly dependent (7–10)	84 (14.7%)
**Number of smokers within five friends, acquaintances or colleagues (*n* = 579)**	
Mean (SD)	3.8 (1.5)
0	42 (7%)
1	76 (12%)
2	130 (22%)
3	124 (21%)
4	125 (21%)
5	82 (14%)

Data are *n* (%) or mean (SD).

**Table 2:** Participant smoking status, partner smoking status, and social support during the period of the smoking cessation program.

Characteristic	Participants
**Participant smoking status ^A^**	
Abstinent after smoking cessation program completion (*n* = 604)	
Yes	482 (80%)
No	122 (20%)
Abstinent 12 months after smoking cessation program completion (*n* = 603) ^B^	
Yes	206 (34%)
No	397 (66%)
**Partner smoking status (*n* = 508)**	
Never smoked	237 (47%)
Continued smoking	108 (21%)
Stopped	63 (12%)
No partner	96 (19%)
Other	4 (1%)
**Group training colleague support (*n* = 503)**	
Not very supportive	112 (22%)
Very supportive	391 (78%)
**Other colleague support (*n* = 500)**	
Not very supportive	304 (61%)
Very supportive	196 (39%)
**Friends and family support (*n* = 509)**	
Not very supportive	247 (49%)
Very supportive	262 (51%)
**Partner support (*n* = 516)**	
Not very supportive	133 (26%)
No partner	106 (20%)
Very supportive	277 (54%)

Data are *n* (%) or mean (SD); ^A^ CO-validated continuous smoking abstinence; ^B^ One participant was excluded from the analysis because of unavoidable loss to follow-up according to the Russell Standard.

**Table 3:** Associations of smoking behavior in the social environment and social support with short- and long-term smoking abstinence.

Variable	Smoking Abstinence Directly after the Smoking Cessation Program ^A^			Smoking Abstinence 12 Months after the Smoking Cessation Program ^B^		
	Odds Ratio	95% Confidence Interval	*p* Value	Odds Ratio	95% Confidence Interval	*p* Value
Number of smokers in close circle (0–5)	0.93	0.80–1.08	0.346	0.81	0.71–0.92	0.002 *
Partner smoking status						
Never smoked (ref.)	1			1		
Continued smoking	0.62	0.33–1.17	0.140	0.40	0.24–0.66	<0.001 *
Stopped	0.87	0.36–2.06	0.745	0.47	0.26–0.86	0.014 *
No partner	0.46	0.25–0.85	0.014 *	0.48	0.26–0.88	0.019 *
Other	0.23	0.09–0.57	0.002 *	0.09	0.02–0.35	<0.001 *
Group training colleague support						
Not very supportive (ref.)	1			1		
Very supportive	3.63	2.07–6.37	<0.001 *	1.85	1.14–3.00	0.013 *
Other colleague support						
Not very supportive (ref.)	1					
Very supportive	0.95	0.57–1.58	0.850	n.s. ^C^	n.s. ^C^	n.s. ^C^
Friends and family support						
Not very supportive (ref.)	1					
Very supportive	1.23	0.76–1.99	0.398	n.s.^C^	n.s. ^C^	n.s. ^C^
Partner support						
Not very supportive or no						
partner (ref.)	1			1		
Very supportive	2.01	1.23–3.30	0.006 *	1.19	0.75–1.88	0.465

The dependent outcome variable was CO-validated continuous smoking abstinence. All analyses were controlled for intervention group, educational level, income level, and Fagerström score. Multiple imputation was used for missing values and a random intercept at company level was used to adjust for the clustering of participants within a company. ^A^ In this analysis, separate logistic regression analyses were used for each individual variable, still including all control variables. ^B^ In this multivariable analysis, a preselection was made first by comparing all variables separately with only the control variables in the model (see Appendix A, multiple imputation). The variables with a P value lower than 0.200 were then selected and included in the multivariable analysis. ^C^ These variables were non-significant in the preselection analysis (see Appendix A, multiple imputation) and therefore not included in the 12-month analysis. * *p* ≤ 0.05.

## Appendix A

**Table A1:** Associations of smoking behavior in the social environment and social support with 12-month smoking cessation: univariable analysis.

Variable	Multiple Imputation			Complete Case Analysis		
	Odds Ratio	95% Confidence Interval	*p* Value	Odds Ratio	95% CI	*p* Value
Number of smokers in close circle (0–5)	0.79	0.69–0.89	<0.001 *	0.80	0.70–0.92	0.002
Partner smoking status						
Never smoked (ref.)	1			1		
Continued smoking	0.36	0.22–0.59	<0.001 *	0.43	0.25–0.73	0.002 *
Stopped	0.47	0.26–0.84	0.012 *	0.53	0.27–1.02	0.059
No partner	0.39	0.23–0.65	<0.001 *	0.37	0.21–0.66	<0.001 *
Other	0.08	0.02–0.33	<0.001 *	0.04	0.01–0.31	0.002 *
Group training colleague support						
Not very supportive (ref.)	1			1		
Very supportive	1.94	1.22–3.10	0.006 *	1.25	0.75–2.07	0.392
Other colleague support						
Not very supportive (ref.)	1			1		
Very supportive	0.83	0.56–1.21	0.328	0.74	0.48–1.14	0.172
Friends and family support						
Not very supportive (ref.)	1			1		
Very supportive	1.11	0.77–1.61	0.566	1.02	0.68–1.54	0.915
Partner support						
Not very supportive or no partner (ref.)	1			1		
Very supportive	1.88	1.30–2.70	>0.001 *	1.66	1.10–2.51	0.016 *

The dependent outcome variable was CO-validated continuous smoking abstinence. Separate logistic regression analyses were used for each individual variable. All analyses were controlled for intervention group, educational level, income level, and Fagerström score, and a random intercept at company level was used to adjust for the clustering of participants within a company. * *p* ≤ 0.05.

## Appendix B

**Table A2:** Associations of smoking behavior in the social environment and social support with short and long-term smoking cessation: complete case analysis.

Variable	Directly after Smoking Cessation Program ^A^			12 Months after Smoking Cessation Program ^B^		
	Odds Ratio	95% Confidence Interval	*p* Value	Odds Ratio	95% Confidence Interval	*p* Value
Number of smokers in close circle (0–5)	0.93	0.79–1.10	0.390	0.81	0.69–0.94	0.006*
Partner smoking status						
Never smoked (ref.)	1			1		
Continued smoking	1.09	0.50–2.37	0.828	0.49	0.28–0.86	0.013 *
Stopped	1.35	0.48–3.83	0.573	0.58	0.29–1.17	0.127
No partner	0.46	0.24–0.89	0.022*	0.46	0.23–0.91	0.027 *
Other	0.39	0.13–1.16	0.090	0.05	0.01–0.38	0.004 *
Group training colleague support						
Not very supportive (ref.)	1			1		
Very supportive	3.53	1.94–6.42	<0.001 *	1.24	0.73–2.11	0.422
Other colleague support						
Not very supportive (ref.)	1					
Very supportive	0.98	0.55–1.73	0.936	n.s.	n.s.	n.s.
Friends and family support						
Not very supportive (ref.)	1					
Very supportive	1.26	0.72–2.20	0.412	n.s.	n.s.	n.s.
Partner support						
Not very supportive or no partner (ref.)	1			1		
Very supportive	2.07	1.17–3.66	0.012 *	1.11	0.66–1.87	0.700

The dependent outcome variable was CO-validated continuous smoking abstinence. All analyses were controlled for intervention group, educational level, income level, and Fagerström score. Multiple imputation was used for missing values and a random intercept at company level was used to adjust for the clustering of participants within a company. ^A^ In this analysis, separate logistic regression analyses were used for each individual variable. ^B^ In this multivariable analysis, all variables were separately assessed with only the control variables in the model (Appendix A, complete case analysis), and then the variables with a P value lower than 0.200 were selected and included in the multivariable analysis. * *p* ≤ 0.05.
